# Magnetic Exchange Mechanism and Quantized Anomalous Hall Effect in Bi_2_Se_3_ Film with a CrWI_6_ Monolayer

**DOI:** 10.3390/molecules29174101

**Published:** 2024-08-29

**Authors:** He Huang, Fan He, Qiya Liu, You Yu, Min Zhang

**Affiliations:** 1 School of Physics and Astronomy, China West Normal University, Nanchong 637002, China; huangh@stu.cwnu.edu.cn (H.H.); hefan@cwnu.edu.cn (F.H.); 2 College of Optoelectronic Engineering, Chengdu University of Information Technology, Chengdu 610225, China; liuqiya@cuit.edu.cn (Q.L.); yy2012@cuit.edu.cn (Y.Y.)

**Keywords:** topological insulator, first principles, magnetic proximity effect, spin

## Abstract

Magnetizing the surface states of topological insulators without damaging their topological features is a crucial step for realizing the quantum anomalous Hall (QAH) effect and remains a challenging task. The TI–ferromagnetic material interface system was constructed and studied by the density functional theory (DFT). A two-dimensional magnetic semiconductor CrWI_6_ has been proven to effectively magnetize topological surface states (TSSs) via the magnetic proximity effect. The non-trivial phase was identified in the Bi_2_Se_3_ (BS) films with six quantum layers (QL) within the CrWI_6_/BS/CrWI_6_ heterostructure. BS thin films exhibit the generation of spin splitting near the TSSs, and a band gap of approximately 2.9 meV is observed at the Γ in the Brillouin zone; by adjusting the interface distance of the heterostructure, we increased the non-trivial band gap to 7.9 meV, indicating that applying external pressure is conducive to realizing the QAH effect. Furthermore, the topological non-triviality of CrWI_6_/6QL-BS/CrWI_6_ is confirmed by the nonzero Chern number. This study furnishes a valuable guideline for the implementation of the QAH effect at elevated temperatures within heterostructures comprising two-dimensional (2D) magnetic monolayers (MLs) and topological insulators.

## 1. Introduction

The discovery of topological insulators (TIs) has spurred significant research interest in condensed matter physics, particularly in exploring exotic phenomena induced by breaking time-reversal symmetry (TRS) and opening surface band gaps [1,2]. In particular, magnetic interactions with topological properties lead to exotic quantum states in materials, including Majorana fermions [3] (non-Abelian statistics) and the quantum anomalous Hall (QAH) effect (dissipationless chiral edge states) [4,5]. Of the two widely employed approaches to break the TRS in TIs, the magnetic proximity effect, achieved through interface formation with a magnetic insulator, has advantages over doping with transition metal atoms into TIs. This is commonly associated with magnetic doping, leading to the formation of defects, including lattice defects, microscopic phase segregation, and impurity bands [6,7]. Following theoretical predictions, the QAH effect was first confirmed in magnetically Cr-doped (Bi, Sb)_2_Te_3_ [5]. Subsequently, researchers observed the QAH effect in various systems, such as Cr-doped (Bi, Sb)_2_Te_3_/Cr_2_O_3_ [8] and a Cr-doped (Bi, Sb)_2_Te_3_/(Bi, Sb)_2_Te_3_ sandwich structure [9]. Despite some work reports on introducing magnetism into Bi_2_Se_3_ (BS), it is still a challenge to achieve complete resistance to the QAH effect due to the influence of the carrier concentration [10,11,12]. Therefore, it is necessary to explore new magnetic introduction methods.

Recently, some theoretical studies have reported on heterojunctions employing magnetic insulator (MI)/TI heterojunctions [13,14,15,16]. Regrettably, in many cases, the hybridization at the interface between an MI and TI is excessively strong, leading to the disruption or misalignment of the topological surface states (TSSs) concerning the Fermi level [17,18]. Theoretical work has proposed that in the heterostructure of three-dimensional topological insulators and magnetic insulators, the magnetic order introduced by the magnetic proximity effect breaks the TRS and exhibits gapped surface states [18,19,20]. Nevertheless, achieving the QAH effect at high temperatures remains a considerable challenge. Ordinarily, the existence of the Mermin–Wagner theorem [21] has led to the belief that establishing a stable magnetic order in two-dimensional materials is impossible. It has been reported that magnetic anisotropy can alleviate this limitation and promote phenomena such as the emergence of two-dimensional (2D) Ising ferromagnetism [22]. Consequently, researchers have unveiled that CrI_3_ is an out-of-plane spin-oriented Ising ferromagnet via magneto-optical Kerr effect microscopy [23], confirming the existence of a 2D Ising magnetic monolayer (ML) in CrI_3_. Due to the weak magnetic coupling [24,25,26] established by super-exchange, ML CrI_3_ exhibits a low Curie temperature (T_c_ ≈ 45 K). Doping metal elements to alloy CrI_3_ is beneficial to enhance its magnetic coupling effect, thereby increasing the Curie temperature. Using alloyed CrI_3_ as a magnetic substrate for topological insulator materials is more conducive to achieving a high-temperature QAH effect.

In our study, a 2D-vdW magnetic semiconductor, CrWI_6_, was created through doping. A CrWI_6_ ML as a semiconductor can maintain the transport properties of TIs, while showing a highly stable magnetic order and significant magnetic anisotropy [23]. Therefore, a CrWI_6_ ML provides an optimal way to magnetize the TSSs of TIs. Additionally, the CrWI_6_/BS/CrWI_6_ heterostructures with different thicknesses of BS thin films were constructed. Based on the density functional theory (DFT), the feasible route to enhance the non-trivial gaps in the heterostructures of utilizing a CrWI_6_ ML to magnetize the TSSs of BS is reported. It is observed that the TSSs of BS can be effectively magnetized by the CrWI_6_ ML. Furthermore, the topological non-triviality of the CrWI_6_/6QL-BS/CrWI_6_ heterostructures was confirmed by the presence of nonzero Chern numbers (C_N_). The experimental observation and practical application of the QAH effect based on the BS/2D-vdW magnetic semiconductor interface system can be further facilitated by our work.

## 2. Model and Computational Details

The DFT calculations used the Vienna Ab Initio Simulation Package (VASP) code [27,28] to study the geometrical and electronic properties of a system. The Perdew–Burke–Ernzerhof (PBE) [29] of the Generalized Gradient Approximation (GGA) [30] exchange–correlation functional method was employed in this study. To better investigate the geometry of heterojunctions, the vdW interaction form of DFT-D3 [31] with Becke–Jonson damping was employed for calculations, as it has been demonstrated to provide a more accurate description for various systems. The plane-wave cutoff energy was set to 520 eV, and the convergence criteria for the total energy and atomic forces were set to be less than 10^−6^ eV and 0.02 eV/Å, respectively. Dudarev’s method [32] was used in all calculations, while the effective Hubbard Ueff 3 eV and 1 eV were added to the Cr-d and W-d orbits, respectively. Spin–orbit coupling (SOC) was also considered, and Brillouin zone (BZ) integration was performed using Gamma-centered 3 × 3 × 1 k-point grids. To avoid spurious interactions due to periodic boundary conditions, a vacuum space of more than 15 Å was used along the *Z* direction.

**Figure 1:**
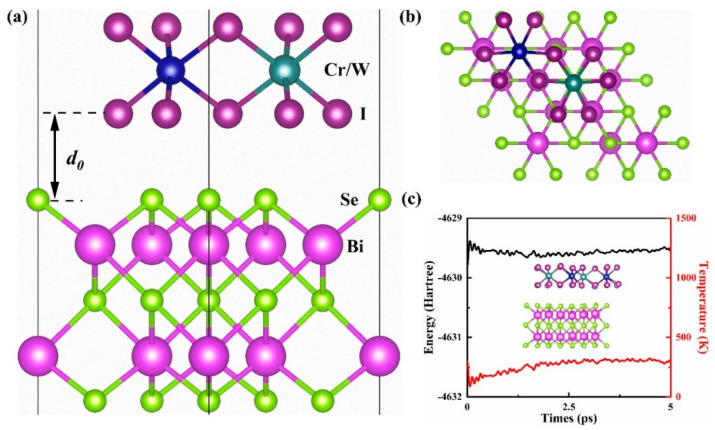
(**a**) Side view of the CrWI_6_/BS interface, *d*_0_ denotes the optimized interlayer distance. The blue atom (left) represent Cr and the green atom (right) represent W. (**b**) Top view of the CrWI_6_/BS interface. (**c**) Energy and temperature fluctuations observed in molecular dynamics simulations of heterojunction interfaces, the inset depicting the final structure.

The present work introduces a theoretical investigation of the magnetic proximity effect in the interface of CrWI_6_/BS (See Figure 1). To check the thermal stability of the interface of the heterojunction, molecular dynamics simulations were carried out using the CP2K 2024.1 software package [33,34], a 4 × 4 × 1 supercell (included 424 atoms) was used, the temperature was maintained at 300 K by using the Nose–Hoover thermostat [35], a time step of 1 femto-second (fs) was used to integrate the equations of motion, and the 5 pico-second (ps) trajectory was generated and used for analysis. There are three positions with high symmetry within the CrWI_6_/BS/CrWI_6_ heterostructure. Among them, the configuration where Cr ions are positioned above Se ions exhibits the lowest energy (see Figure S1). Therefore, unless otherwise specified in the following sections, we adopt the heterostructure model where Cr ions are positioned above Se ions. The interlayer distance between the heterojunctions and calculation parameters were determined through an energy test (see Figure S2); the results indicated that the optimal interlayer distance, denoted as d_0_, for the CrWI_6_/BS/CrWI_6_ heterojunction is 3.18 Å. Further stability calculations confirmed that d_0_ remains unchanged after the relaxation of the structure, demonstrating its structural stability.

## 3. Results and Discussion

### 3.1. Magnetism and Structure of CrWI_6_

**Figure 2:**
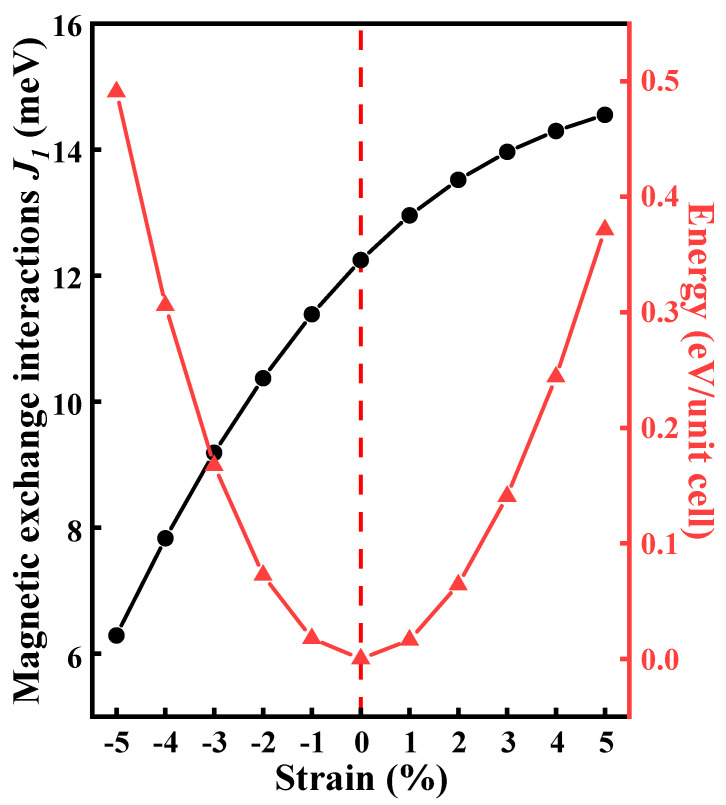
The influence of biaxial strain on the nearest-neighbor magnetic exchange coupling effect and energy transformation trend of CrWI_6_.

To check the dynamical stabilities of the CrWI_6_ ML, the phonon spectra are computed using density functional perturbation theory (DFPT) [36]. The phonon dispersion relation is obtained by processing the data with the PHONOPY 2.26.0 software package [37], as illustrated in Figure S3. After structural optimization, it was observed that the lattice constant of the CrWI_6_ ML measures 6.94 Å, representing a diminution of merely 3.9% in comparison to the lattice constant of the √3 × √3 BS supercell (7.22 Å). Additionally, the application of biaxial strain (−5~5%) revealed that the magnetic properties of the CrWI_6_ ML remain unaffected in the presence of minor lattice stretching (see Figure 2).

The presence of magnetic anisotropy is a crucial requirement for the existence of long-range magnetic ordering in two-dimensional systems [38]. To assess the magnetic anisotropy energy (MAE) in the single-layer CrWI_6_ with spin orientation along different directions, the angular dependence of the MAE was determined using the following equation [39]:

(1) $$\mathrm{MAE}\left(\theta ,\varphi \right)=E\left(\theta ,\varphi \right)-E\left(\theta =0,\varphi =0\right)$$

Here, θ represents the angle between the magnetization direction and the *Z*-axis, while φ denotes the angle between the projection of the magnetization direction on the *XY*-plane and the *X*-axis. The calculation results reveal that the magnetic easy axis of CrWI_6_ ML is perpendicular to the *XY*-plane, which means that the easy magnetization axis consistently aligns with the *Z*-axis, and it can effectively magnetize the BS film peeled from the BS 001 crystal plane. In two-dimensional magnetic materials, the magnetic anisotropy of CrWI_6_ is relatively more significant [40]. This unique characteristic is predicted to play a crucial role in stabilizing long-range magnetic coupling by offering resistance against thermal perturbations (See Figure S4). The substantial MAE observed in CrWI_6_ is indicative of a remarkably robust magnetic coupling interaction within the material. This is further highlighted by its ability to maintain magnetic order even at elevated temperatures. To quantify this behavior, the T*_c_* of CrWI_6_ was determined through Monte Carlo simulations applied to the Heisenberg model:

(2) $$H=\sum_{ij}J_{ij}S_{i}S_{j}$$

**Figure 3:**
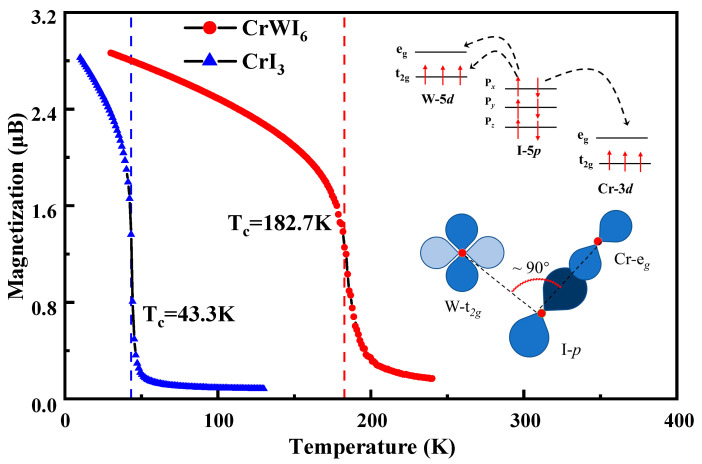
Based on the Heisenberg model, the T*_c_* of CrWI_6_ and CrI_3_ are computed through the application of Monte Carlo simulation. Schematic diagrams of the super-exchange interaction and FM coupling in CrI_3_ and CrWI_6_ ML.

Generally, considering only the exchange interaction between the nearest and next-nearest neighbors is sufficient to describe the magnetic interactions in magnetic systems [41], where J_ij_ represents the nearest and the next-nearest-neighbor exchange interactions, and S_i_ represents the spin at site i, respectively. For CrWI_6_ ML, our results demonstrate that the establishment of the ferromagnetic (FM) ground state is mainly governed by the nearest-neighbor exchange interaction (J_1_). Additionally, the next-nearest-neighbor interactions (J_2_) also favor FM ordering; notably, due to an order of magnitude difference, we exclusively considered the magnetic coupling constants J_1_ and J_2_, excluding J_3_. The T*_c_* is taken as the critical point of the specific heat. As shown in Figure 3, the simulation results indicate that the T*_c_* transition for CrI_3_ is at 43.3 K (very close to the experimental value ~45 K [42]), thereby validating the reliability of our simulation. In contrast, CrWI_6_ ML exhibits the T*_c_* of 182.7 K, FM super-exchange interactions in CrWI_6_ ML via the e*_g_*-(p*_x_*, p*_y_*, p*_z_*)-t*_2g_* orbitals, and the Cr-I-W bonding angle is close to 90 degrees, which explains the occurrence of FM coupling according to the well-known Goodenough−Kanamori−Anderson (GKA) rules [24,25,26] of the super-exchange theorem. Given the elevated T*_c_* observed in CrWI_6_, the formation of a heterojunction with the BS becomes a feasible prospect, potentially enabling the realization of the QAH effect at a higher temperature.

### 3.2. Electronic Properties of Heterojunction

To clarify the interactions at the interface, the spin density Δσ and charge density difference Δρ at the CrWI_6_/6QL-BS interface were plotted, which is defined as follows

(3) $$\Delta \sigma =\sigma_{↑}-\sigma_{↓}$$

(4) $$\Delta \rho =\rho_{total}-\rho_{CrWI6}-\rho_{BS}$$

**Figure 4:**
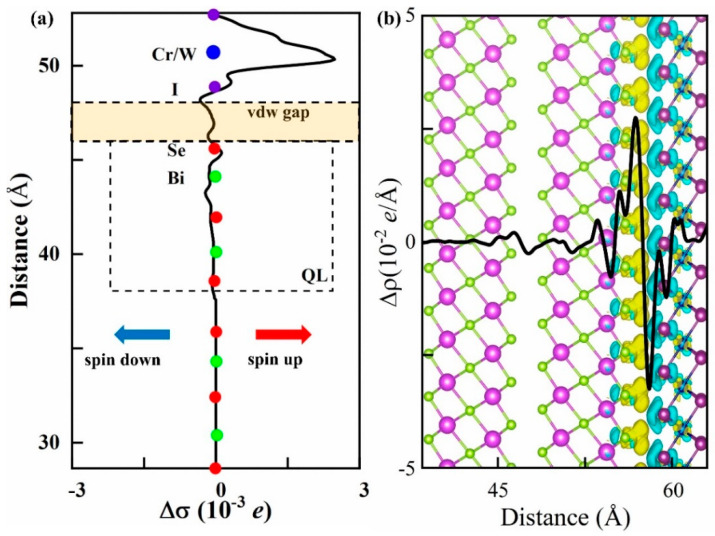
(**a**) Planar-averaged spin density Δσ in the interfacial region of the interface of the heterostructure. (**b**) The distribution of the charge difference Δρ.

Here, $\sigma_{↑}$ and $\sigma_{↓}$ denote the spin-up electrons and spin-down electrons, and $\rho_{total}$, $\rho_{CrWI6},$ and $\rho_{BS}$ were the charge densities of the interface of the heterojunction, CrWI_6_ ML, and the BS film, respectively, as shown in Figure 4. Similar to prior studies [20,43], the magnetic proximity effect is prominent in the QL nearest to the interface. The Se atomic layer of BS closest to the interface acquires a small but non-negligible magnetic moment, aligning with the Cr/W ion spin in the CrWI_6_ ML. To further discuss the origin of the magnetic proximity effect, the charge differential density at the interface of the heterojunction was analyzed. The charge transfer of the interface of the heterojunction predominantly takes place at the interface between CrWI_6_ and BS; the positive values (yellow area) indicate electron accumulation, and the negative values (cyan area) represent electron depletion. Additionally, the plane-averaged charge density difference at the interface is illustrated. Regions where Δρ is less than zero indicate electron dissipation, whereas values greater than zero signify electron accumulation. Consequently, it is evident that within the CrWI_6_ ML, electrons are transferred from I to Cr/W atoms. At the interface, the electron transfer occurs from I to Se-p orbit, providing an explanation for the spin-up magnetic moment of Se atoms.

**Figure 5:**
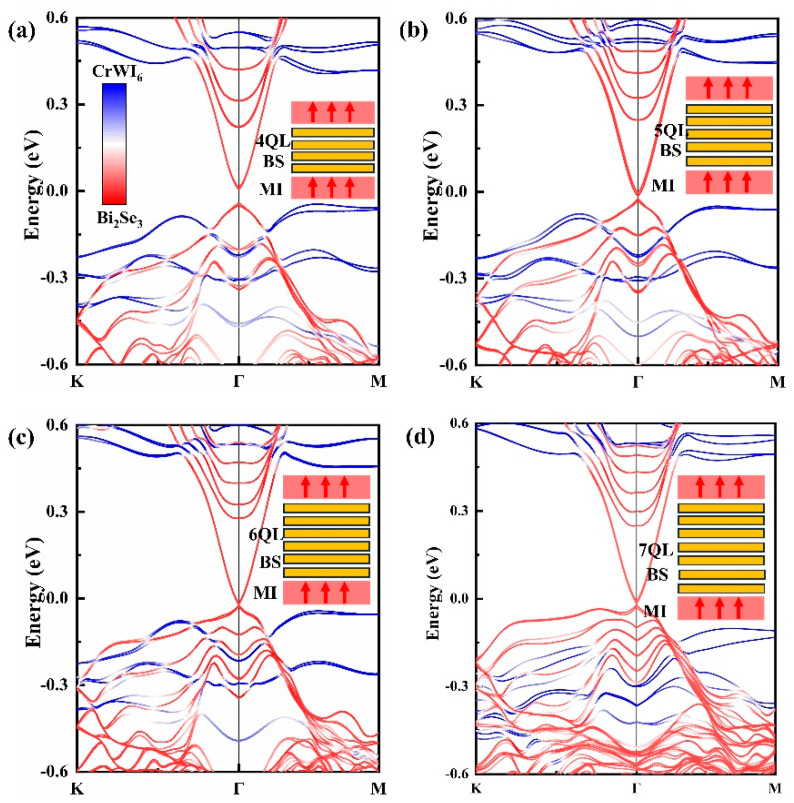
Band structures of CrWI_6_/BS/CrWI_6:_ (**a**) CrWI_6_/4QL-BS/CrWI_6_, (**b**) CrWI_6_/5QL-BS/CrWI_6_, (**c**) CrWI_6_/6QL-BS/CrWI_6,_ and (**d**) CrWI_6_/7QL-BS/CrWI_6_. Colors in the main panels indicate the weights of bands from BS (red) and CrWI_6_ (yellow).

For BS, a reduction in the number of QLs results in the hybridization and annihilation of TSSs on both the upper and lower surfaces of the TI [44]. Hence, the heterojunction consists of 4, 5, and 6 QLs of BS and CrWI_6_. Through the projected band structure analysis (See Figure 5), the band contributions in proximity to the Fermi level at the heterojunction were ascertained. The energy band near the Dirac cone is primarily composed of BS, while the contribution from CrWI_6_ is concentrated around 0.5 eV and −0.2 eV, the same as with the energy band structure of intrinsic CrWI_6_ (See Figure S5), and the TSSs near the Dirac cone are almost unaffected by CrWI_6_. These results show that 2D-vdW magnetic semiconductors are more suitable for the magnetization of TSS than other ferromagnetic or antiferromagnetic films.

**Figure 6:**
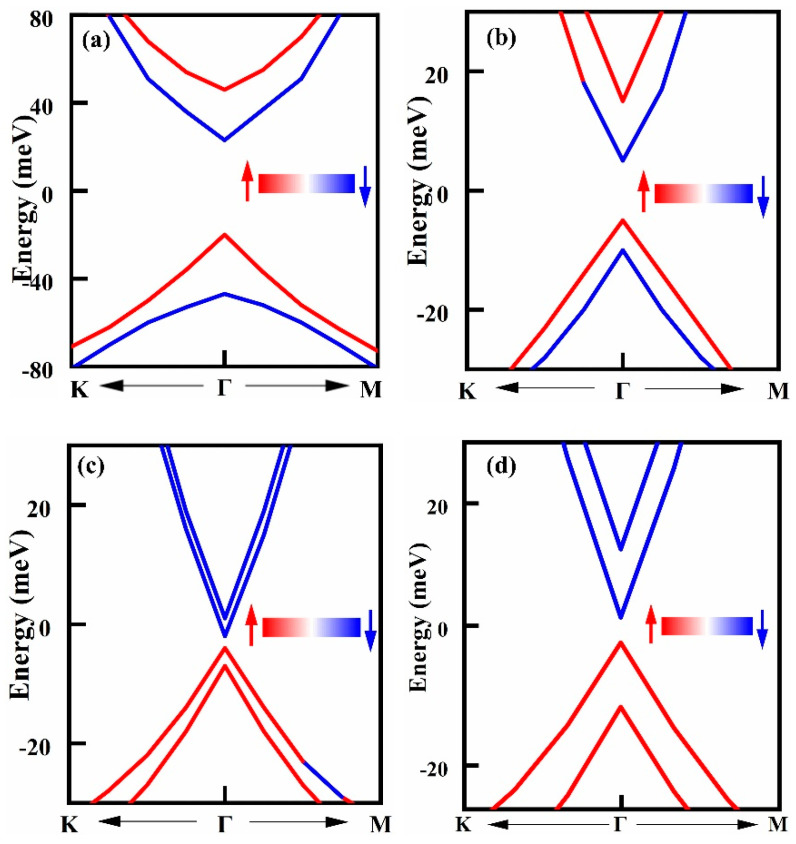
Spin projections of CrWI_6_/BS/CrWI_6_ band structure: (**a**) CrWI_6_/4QL-BS/CrWI_6_, (**b**) CrWI_6_/5QL-BS/CrWI_6_, (**c**) CrWI_6_/6QL-BS/CrWI_6,_ and (**d**) CrWI_6_/7QL-BS/CrWI_6_ The red arrow indicates spin-up electrons and the blue arrow indicates spin-down electrons.

To reveal the successful magnetization of TSSs, the spin projections of the band structure near the Dirac cone in the 4–6 QLs energy band are plotted, with the red arrow indicating spin-up electrons and the blue arrow indicating spin-down electrons. It is observed that the spin degeneracy of TSSs near the Fermi level is disrupted in all cases (See Figure 6). Upon reaching a 6QL of the BS film, the band composition near the Fermi level undergoes a change. There is a band gap of approximately 2.9 meV near the Dirac cone. Specifically, in the 6QL-BS/CrWI6 system, the valence band is composed of spin-up components, while the conduction band is composed of spin-down components. In contrast, for BS systems with fewer than 5QLs, both the valence band and conduction band contain spin-up and spin-down components. The significant difference in the energy band composition at this point indicates that the system is transitioning from a normal insulator to a Chern insulator state, signifying that the system is topologically non-trivial [43].

To verify the potential influence of different interface stacking manners on the results, we computed energy bands for two additional stacking manners. The spin projections for the two alternative stacking manners in CrWI_6_/6QL-BS/CrWI_6_ are shown in Figure S6. The results indicate that different stacking manners do indeed affect the band gaps. The stacking configuration of Cr/W atoms above Se atoms exhibits the largest non-trivial band gaps, possibly due to the lowest energy associated with this stacking mode. However, due to the six QLs of BS, the TSSs of this system remain stable, and thus, the non-trivial band structure is preserved. The energy gap data can be found in Table 1.

### 3.3. Topological Properties

To assess the topological features of the magnetized TSSs of CrWI_6_/6QL-BS/CrWI_6_, a tight-binding model system based on Wannier functions was constructed. Using this model, the band structure and Berry phase by integrating the Berry curvature over the BZ were calculated. The VASPBERRY package [45] was used to perform calculations for the Berry curvature and Chern numbers of BZ, and the Berry curvature Ω(k) is defined by the following equation [46,47]:

(5) Ωk=∑nfnΩnk=−∑n′≠n2Imψnkvxψn′kψn′kvyψnkεn′k−εnk

(6) $$C_{N}=\underset{n\in \left\{0\right\}}{\sum }C_{n}=\frac{1}{2\pi }\int \underset{n\in \left\{0\right\}}{\sum }\Omega_{n}\left(k\right)d^{2}k=\underset{n\in \left\{0\right\}}{\sum }\left(C_{n,↑}+C_{n,↓}\right)$$

Here, $f_{n}$ is the Fermi distribution, $\psi_{nk}$ is the eigenstate of the wave function, $v_{x}$ and $v_{y}$ are the velocity operator, and $\left\{0\right\}$ represents the occupied state. The anomalous Hall conductance around the Fermi level is defined by the following equation:

(7) $$\sigma_{xy}=\frac{e^{2}}{h}C_{N}$$

**Figure 7:**
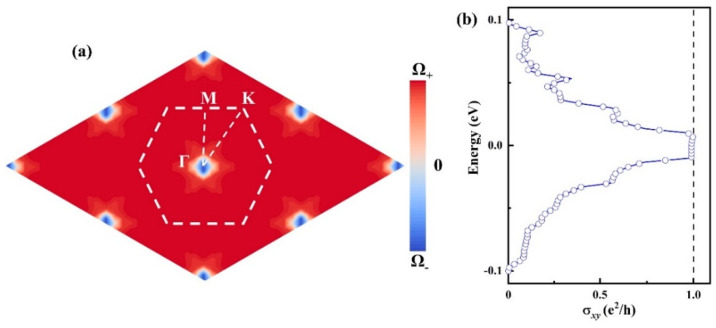
(**a**) Berry curvature within the SOC gap in reciprocal space of CrWI_6_/6QL-BS/CrWI_6_. (**b**) Hall conductivity of CrWI_6_/6QL-BS/CrWI_6_.

Firstly, utilizing the above formula, the distribution of the Berry curvature for the valence band of the CrWI_6_/6QL-BS/CrWI_6_ system was computed, as depicted in Figure 7a. By integrating the Berry curvature, a non-trivial Chern number $C_{N\;}=1$ was determined. Within the energy window of the SOC gap, one can observe a quantized Hall plateau at a value of $e^{2}/h$, as illustrated in Figure 7b.

**Figure 8:**
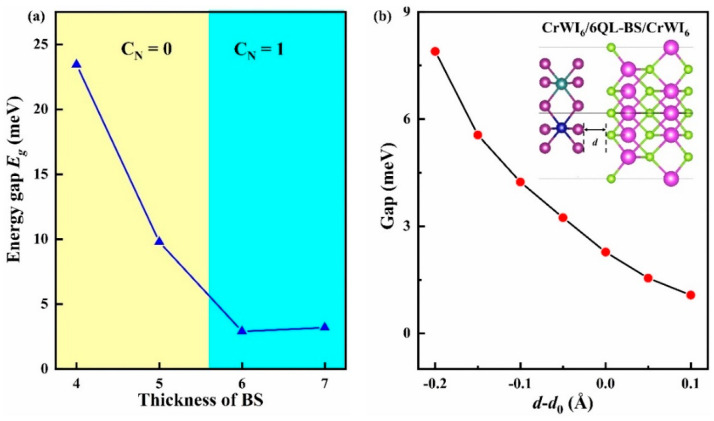
(**a**) Dependence of Chern numbers and gaps of CrWI_6_/BS/CrWI_6_ on the QL of BS film. (**b**) The impact of the interlayer distance on the gap of CrWI_6_/6QL-BS/CrWI_6_.

Secondly, by computing the CrWI_6_/BS/CrWI_6_ heterostructure with different layer thicknesses of BS, we summarized the trends of the band gaps and Chern numbers for the CrWI_6_/BS/CrWI_6_ heterostructure at different layers, as shown in Figure 8a. The calculated gaps of CrWI_6_/6QL-BS/CrWI_6_ and CrWI_6_/7QL-BS/CrWI_6_ are 2.3 meV (26.7 K) and 3.2 meV (37.1 K), respectively. Therefore, it can be concluded that a CrWI_6_/BS/CrWI_6_ heterostructure with fewer than six QLs of BS behaves as a normal insulator. Considering that the T_c_ of CrWI_6_ ML is 183 K, and that the heterostructure system does not involve other complex factors such as uncontrollable doping distribution and local magnetic ordering, the QAH effect should be observed in the heterostructure at temperatures as high as several tens of Kelvin.

Finally, although the non-trivial band gaps in the CrWI_6_/6QL-BS/CrWI_6_ heterostructure can reach 2.9 meV, they are still relatively small compared to similar systems with the same number of BS layers, such as Cr_2_Ge_2_Te_6_/Bi_2_Se_3_/Cr_2_Ge_2_Te_6_ 19.5 meV [48] and MnBi_2_Te_4_ 33 meV [49]. To increase the system’s non-trivial band gaps and enhance the temperature at which the QAHE is observed, we simulated external pressure by adjusting the interface distance d of CrWI_6_/6QL-BS/CrWI_6_ to compress the heterostructure. The results are shown in Figure 8b. The results indicate that reducing the interlayer distance can effectively widen the band gaps, facilitating the realization of the QAH effect. When reducing the interface distance to simulate external compression in the vertical direction, the band gaps of the CrWI_6_/6QL-BS/CrWI_6_ heterostructure can reach 7.9 meV (91.6 K); it is indicated that it is beneficial to apply external pressure to reduce *d* for the realization of the QAH effect.

## 4. Conclusions

In summary, this study has explored the feasibility of achieving the QAH effect in a TI–ferromagnetic material interface system composed of BS and CrWI_6_ at higher temperatures. Through DFT to investigate the electron features, we have demonstrated that the 2D-vdW, a 2D-vdW magnetic semiconductor, can effectively magnetize the TSSs of TIs while preserving their topological features around the Fermi level. The topological non-trivial features of the CrWI_6_/6QL-BS/CrWI_6_ interface were verified by nonzero Chern numbers. There was a band gap of approximately 2.9 meV near the Dirac cone. By adjusting the interface distance of the heterostructure, we increased the non-trivial band gap to 7.9 meV, indicating that applying external pressure is conducive to realizing the QAH effect. This strongly indicates the significant possibility of detecting an induced QAH effect in the experiment. Consequently, this research has the potential to advance the experimental observation and practical utilization of the QAH effect in the TI-MI interface system.

## Figures and Tables

**Table 1:** Energy gaps *E_g_* of various thickness CrWI_6_/BS/CrWI_6_ heterostructures.

Thickness of BS	Energy Gaps *E_g_* (meV)
4QLs	23.5 (Se site)
5QLs	9.8 (Se site)
6QLs	2.9 (Se site) 1.4 (Bi site) 1.1 (Hole site)
7QLs	3.2 (Se site)

## Data Availability

No new data were created or analyzed in this study. Data sharing is not applicable to this article.

## Supplementary Materials

The following supporting information can be downloaded at: https://www.mdpi.com/article/10.3390/molecules29174101/s1.
