# Supplementary material for: Magnetic Exchange Mechanism and Quantized Anomalous Hall Effect in Bi2Se3 Film with a CrWI6 Monolayer
Source: Molecules. 2024 Aug 29;29(17):4101. doi: 10.3390/molecules29174101 (PMC11396793; doi:10.3390/molecules29174101)
Supplement: Supplementary file 1 [file molecules-29-04101-s001.zip › molecules-3131013-supplementary.pdf]

Supporting Information

Magnetic exchange mechanism and quantized anomalous Hall effect in Bi_2Se_3 film with a $CrWI_6$ monolayer

He Huang [a], Fan He [a], Qiya Liu [b], You Yu [b], Min Zhang [a*]

[a] *school of Physics and Astronomy, China West Normal University, Nanchong 637002, China*：zmzmi1987@cwnu.edu.cn

[b] *College of Optoelectronic Engineering, Chengdu University of Information Technology, Chengdu 610225, China*

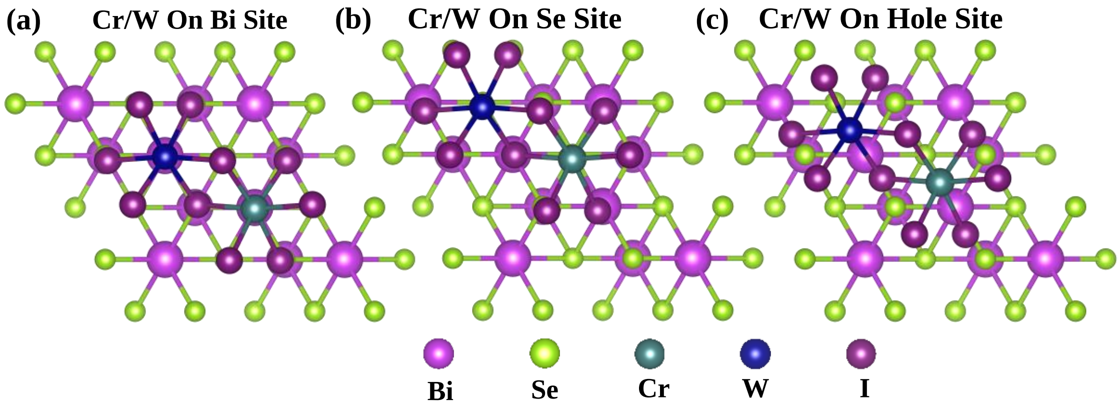

Figure S1. Top views of three sites between $CrWI_6$ and BS in CrI_6/BS/CrI_6 heterostructures. (a) The Cr/W atom in $CrWI_6$ is located above the Bi atom in Bi_2Se_3. (b) The Cr/W atom in $CrWI_6$ is located above the Se atom in Bi_2Se_3. (c) The Cr/W atom in $CrWI_6$ is located above the hole site in Bi_2Se_3.

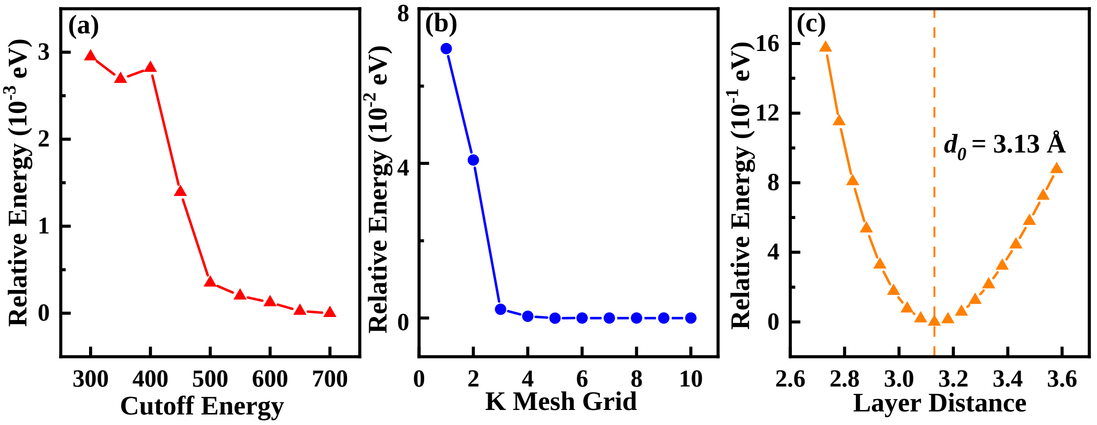

Figure S2. Convergence analysis for the determination of computational parameters (a) Cutoff energy. (b) K-point grid. (c) Heterojunction layer distance.

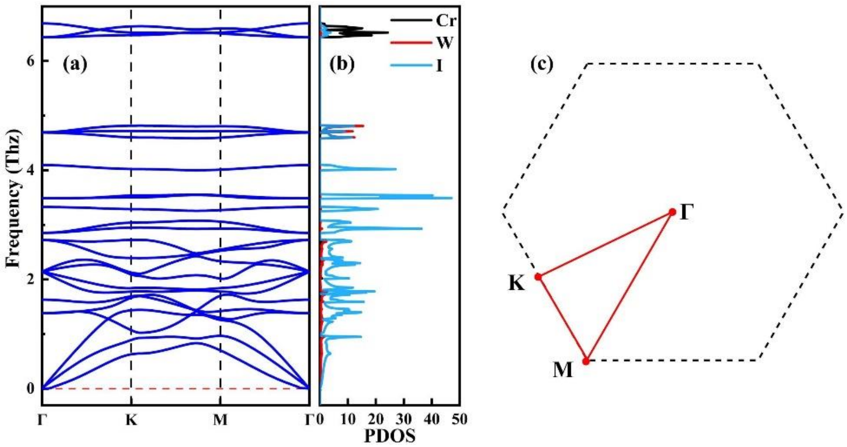

Figure S3. (a) Phonon dispersion curves and (b) phonon density of states of the $CrWI_6$ ML. (c) The high symmetry path created within the BZ.

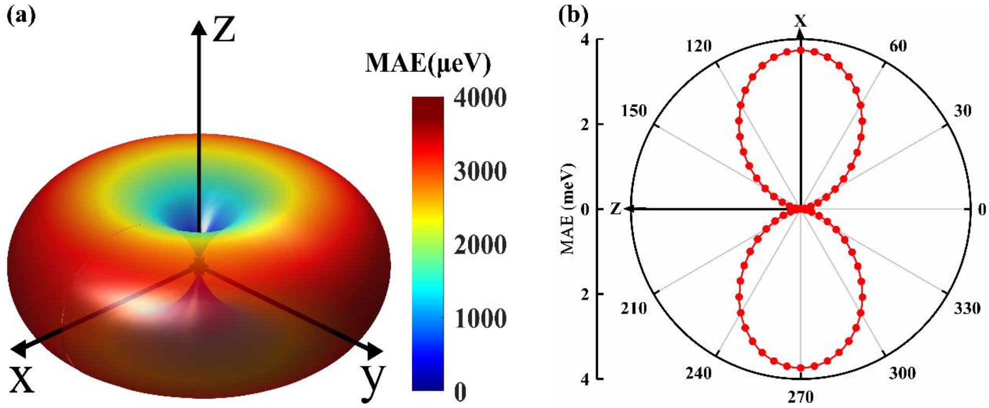

Figure S4. (a) Schematic depiction showcasing the magnetic anisotropy energy (MAE) characteristics of $CrWI_6$ within a three-dimensional spatial framework. (b) Projection of the MAE onto the XZ plane.

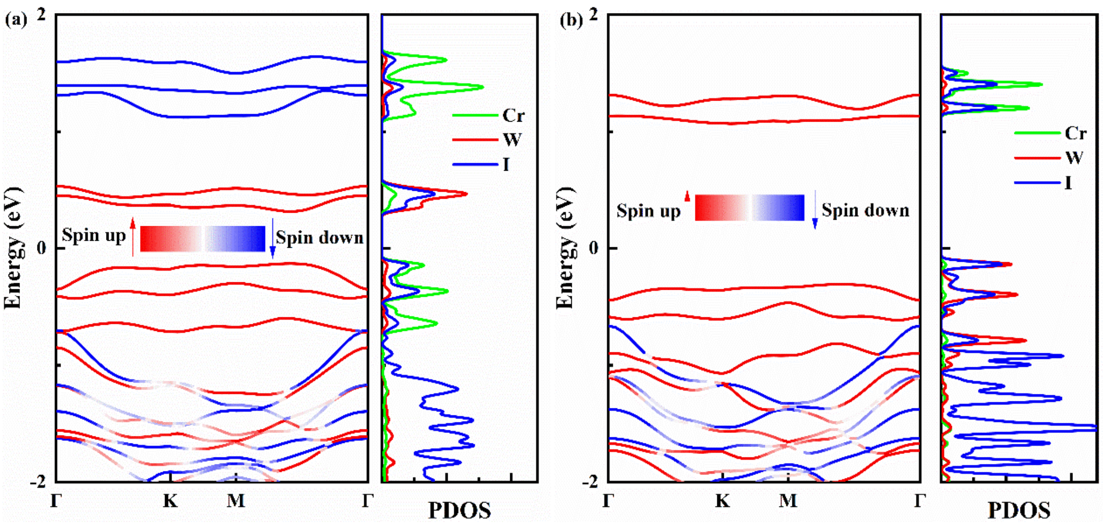

Figure S5. The band structures and partial density of states of monolayer $CrWI_6$ obtained by (a) PBE method and (b) HSE06 method.

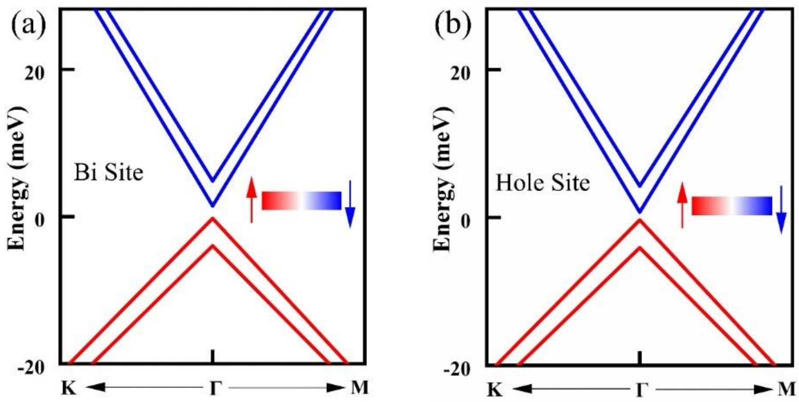

Figure S6. The spin projections of $CrWI_6$/6QL-BS/$CrWI_6$. (a) Bi site (b) hole site. The red arrow indicating spin up electrons and the blue arrow indicating spin down electrons.
